# Cross-Frequency Coupling in Developmental Perspective

**DOI:** 10.3389/fnhum.2019.00158

**Published:** 2019-05-14

**Authors:** Gennady G. Knyazev, Alexander N. Savostyanov, Andrey V. Bocharov, Sergey S. Tamozhnikov, Elena A. Kozlova, Irina V. Leto, Helena R. Slobodskaya

**Affiliations:** ^1^Laboratory of Psychophysiology of Individual Differences, Institute of Physiology and Basic Medicine, Novosibirsk, Russia; ^2^Humanitarian Department, Novosibirsk State University, Novosibirsk, Russia

**Keywords:** cross-frequency coupling, EEG, developmental trends, introversion, social anxiety, source-level analysis, growth curve analysis

## Abstract

It is generally assumed that different electroencephalogram (EEG) frequency bands are somehow related to different computational modes in the brain. Integration of these computational modes is reflected in the phenomenon of cross-frequency coupling (CFC). On slow temporal scales, CFC may reflect trait-like properties, which posits a question of its developmental trends. This is the first study that explored source-level CFC measures in a developmental perspective using both cross-sectional and longitudinal designs. CFC measures demonstrated good test-retest stability and proved to be higher in adults in cortical areas participating in sensory-motor integration, response inhibition, and attentional control. In children, greater CFC was observed in parietal regions involved in self-centered cognition. Over the period from 7 to 10 years, CFC demonstrated nonlinear growth trajectories. Introversion was associated with higher CFC in cortical areas related to emotion, attention, and social cognition, implying that the association between introversion and CFC appears early in the development.

## Introduction

A growing evidence supports the view that oscillations in different electroencephalogram (EEG) frequency bands are associated with different kinds of computation in the brain (Nunez, 2000; Buzsáki and Draguhn, 2004; Engel et al., 2013; Lopes da Silva, 2013). The segregation of cognitive domains associated with different frequency oscillations must be accompanied by their integration, otherwise, brain functioning would not be efficient (Deco et al., 2015). This integration could be reflected in the phenomenon of cross-frequency coupling (CFC). In spite of the steady growth of interest related to this phenomenon, its functional significance is still under debate.

CFC is an umbrella term for a handful of different phenomena related to interactions between different frequency oscillations. Currently, most interest is attracted to CFC at millisecond temporal scales, such as phase-amplitude coupling (PAC) and amplitude envelope coupling (AEC), whose involvement in sensory perception and memory encoding has been repeatedly demonstrated (Tort et al., 2009; Axmacher et al., 2010; Canolty and Knight, 2010; Spaak et al., 2012; Lisman and Jensen, 2013). Another approach to the study of CFC is to measure correlations between amplitude or power of different frequency oscillations at lower temporal scales (usually several seconds) or in the between-subject domain (see Schutter and Knyazev, 2012 for a review). These correlations may reflect some more stable processes, related to emotional states or even traits. Recently, correlations between delta and theta on the one hand and alpha and beta on the other has attracted attention of researchers as a potential marker of aversive states. Much evidence links low frequency oscillations of delta and theta ranges with motivational and emotional processes; alpha and beta oscillations, but on the other hand, are associated with attentional control and regulation of negative affect (Klimesch, 1999; Aftanas et al., 2001; Knyazev, 2007, 2012, 2013; Güntekin and Başar, 2010; Spaak et al., 2012; Jensen et al., 2014; Knyazev et al., 2015). The slow-fast wave (S-F) coupling was measured by correlating powers of respective frequency bands either in between- (i.e., across subjects) or within- (i.e., across data epochs) subject domains in resting condition, or in a state of enhanced anxiety (e.g., Knyazev et al., 2006; Harrewijn et al., 2018), or after administration of hormones (Schutter and van Honk, 2004, 2005; Schutter et al., 2006; Rutherford et al., 2018). The correlations were measured between delta and alpha (e.g., Knyazev and Slobodskaya, 2003; Rutherford et al., 2018), delta and beta (e.g., Schutter and van Honk, 2004, 2005; Velikova et al., 2010; Harrewijn et al., 2018), theta and alpha (e.g., Rutherford et al., 2018), and theta and beta (Schutter et al., 2006; Rutherford et al., 2018). Enhanced S-F coupling was most frequently observed in subjects with social anxiety (Miskovic et al., 2010, 2011b; Harrewijn et al., 2016, 2017, 2018), high scores on behavioral inhibition (Knyazev and Slobodskaya, 2003; Putman, 2011; Putman et al., 2012) or state anxiety (Knyazev, 2011), high salivary cortisol levels (Schutter and van Honk, 2005), and in experimental situations modeling the state of anxious anticipation (Knyazev et al., 2005, 2006; Harrewijn et al., 2018). Most of these studies used adult samples. Information on S-F coupling in children is limited. It has been shown that the strength of relationship between alpha and delta is lower in children than in adults (Knyazev et al., 2003, 2004) and within-subject measures of S-F coupling were differently associated with behavioral inhibition scores in adults and adolescents (Knyazev and Slobodskaya, 2003). Miskovic et al. (2011a) revealed increased frontal S-F coupling in children of parents with social phobia.

Thus, a systematic investigation of developmental changes in S-F coupling is lacking in the literature. Moreover, an overwhelming majority of published studies used channel-level analysis of S-F coupling with a limited number of channels, typically two frontal, two central, and two parietal channels (but see Velikova et al., 2010; Knyazev, 2011 for an exception). In this study, we aimed to perform source-level analysis of S-F coupling in children and adults using both cross-sectional and longitudinal designs. Although many studies highlight that S-F coupling is state-dependent, many other studies reveal its trait-like properties (e.g., its correlation with psychopathology or personality variables). In this study, we did not aim to manipulate the subject’s state and obtained S-F coupling measures in a resting condition with eyes closed and open. We also obtained self-report measures of personality in adults and parental ratings of personality in children. We expected that S-F coupling would be stronger in adults than in children and would increase in children during development. We also expected that measures of S-F coupling would correlate with personality traits related to social anxiety (i.e., introversion and neuroticism).

## Materials and Methods

### Participants

The cross-sectional sample included 170 children from 6 to 11.5 years (93 boys; mean age = 8.5, SD = 1.4). The adult sample included 115 graduate and postgraduate students and staff members of Novosibirsk State University (41 men; mean age = 25.5, SD = 9.2). All participants had no history of neurological, psychiatric, or major somatic disorders.

The longitudinal sample included 68 children (41 boys), who were a part of the cross-sectional sample. EEG data were collected in four consecutive years, approximately in the middle of a respective school year. In the first wave, all children were first-graders. Not all children were available in all four waves. Only children who accomplished at least two visits were included in the analysis. Table 1 shows the composition of the sample in the four waves of the study.

**Table 1:** The distribution of children across longitudinal and cross-sectional samples.

	Wave 1	Wave 2	Wave 3	Wave 4	Cross-sectional
Age (years)	7.0	8.4	9.4	10.6	8.4
*N*	68	43	57	35	170
Boys	41	27	33	20	93

All subject protection guidelines and regulations were followed in accordance with the Declaration of Helsinki. The study aim was explained to all adult participants and all parents and they signed the informed consent. The study and the consent form were approved by the Institute of Physiology and Basic Medicine ethics committee.

### Procedure

EEG was recorded in a soundproof dimly illuminated room. Participants were asked to minimize movement and blinking. Six 1-min recordings with eyes closed and six 1-min recordings with eyes open alternated sequentially. In the eyes open condition, participants were asked to look at an empty monitor screen situated at a distance of 120 cm from the participant.

### Psychometric Instruments

Parents of all children completed a short version of the Inventory of Child Individual Differences (ICID-S, Slobodskaya and Zupancic, 2010), which was developed for the assessment of child personality in terms of the Five-Factor Model (Halverson et al., 2003). The ICID-S includes 62 items rated on a 7-point scale ranging from “much less than the average child” to “much more than the average child.” The items represent 15 lower-order traits that form the five higher-order factors (Halverson et al., 2003); higher scores indicated higher levels of personality traits. Extraversion comprises of activity level (energetic, active physically), positive emotions (sweet, loving) and sociable (likes to be with people). Disagreeableness includes antagonism (confrontational behavior), strong willed (bossy, self-assertive) and reversed considerate (concerned about others). Conscientiousness comprises of achievement orientation (self-discipline and focus on goal attainment), compliant (cooperative in response to authority), organized (orderly and tidy) and reversed distractible (poor concentration, short attention span). Neuroticism includes fearful (insecure, lacks confidence), shy (socially reticent) and negative affect (irritable, quick-tempered). Openness comprises of intelligent/quick to learn (good memory and thinking abilities), and open to experience (imagination, tendency to explore). The Russian version has been validated, supporting good and validity of the scales (Slobodskaya and Zupancic, 2010). In the present study, all scales showed good internal consistency reliability: Cronbach’s alphas were 0.82 for extraversion, 0.88 for agreeableness, 0.90 for conscientiousness, 0.85 for neuroticism, and 0.87 for openness. For mean (SD) scores of ICID-S scales in a normative sample please see Slobodskaya and Zupancic (2010).

### EEG Recording and Preprocessing

In children, EEG was recorded by means of 64 electrodes mounted in an Easycap of appropriate size and referenced to vertex. In adults, a Quik-Cap128 NSL with 118 electrodes was used for EEG registration, but only signals from the 64 electrodes were used in the analysis for the sake of consistency. The electrooculogram was also recorded. “Neuroscan (USA)” amplifiers with 0.1–100 Hz analog band-pass filter were used for signal amplification. The sampling rate was 1,000 Hz. FASTRAK digitizer (Polhemus) was used to measure the actual position of each electrode and five fiduciary points (nasion, inion, two preauricular points, and vertex). Electrode impedances were at or below 5 kilo-ohms. Artifacts were eliminated by means of independent component analysis in EEGlab toolbox^1^. The number of rejected components was 7.4 on average and was not significantly different in children and adults and in children’s recordings in different time points. It did not correlate with psychometric variables. Artifact-corrected EEG was down-sampled and re-referenced to average reference.

### EEG Data Reduction

Eyes-closed and eyes-open continuous EEG recordings were divided into epochs of 4 s length and combined together. The number of epochs ranged from 150 to 180 with no difference between adults and children and between EEGs obtained in different time points. sLORETA was used for inverse source reconstruction. The transformation matrix was constructed for each dataset individually based on the measured electrode positions and the five fiduciary points. The source space consisted of 6,430 voxels at 5-mm spatial resolution within the cortical gray matter and the hippocampus (Pascual-Marqui, 2002). We used the regularization factor 1/100 (Congedo, 2006). Source reconstruction was performed in the frequency domain based on EEG cross-spectra. Because EEG bands’ boundaries change in the course of maturation (Klimesch, 1999; Niedermeyer and Da Silva, 2004), they were determined separately for each participant using individual alpha peak frequency as the anchor point (Doppelmayr et al., 1998). We used the methodology described in Lansbergen et al. (2011), which gives a more accurate estimation of individual alpha peak frequency (IAF) than traditional methods. First, frequency of the maximal alpha power was determined at parietal and occipital channels in the eyes closed condition. Then, EEG spectrum data in the eyes open condition were subtracted from the EEG spectrum data in the eyes closed condition, which allowed to find the frequency at which alpha power was most attenuated upon eyes opening (Klimesch, 1999). If the two peak frequencies did not differ more than 0.5 Hz, the result of the second method was used as individual alpha peak frequency (IAF). Otherwise, IAF was determined by visual inspection of the EEG spectrum in the eyes closed condition. The bandwidth for delta, theta, alpha, and beta were defined as 1–0.4*IAF, 0.4*IAF–0.8*IAF, 0.8*IAF–1.2*IAF, and 1.2*IAF–25, respectively. Subsequently, for each participant separately, S-F Pearson correlations were calculated across the data epochs for each voxel and Fisher Z-transformed. The following correlations were calculated: delta-alpha, delta-beta, theta-alpha, and theta-beta. Additionally, mean S-F correlations were calculated as mean delta and theta vs. mean alpha and beta. The obtained correlation maps were used in subsequent second-level statistical analyses.

### Statistical Analysis

Cross-sectional analyses were performed using mass-univariate statistical nonparametric mapping in sLORETA package. This method is not based on distributional assumptions and is effective for controlling Type I error (Flor-Henry et al., 2004). Longitudinal data were analyzed using the method proposed by Guillaume et al. (2014). The false discovery rate (FDR) correction was used to control for multiple comparison problem. Non-parametric permutation inference was performed using the Wild Bootstrap method, which is appropriate for the analysis of longitudinal data (Liu and Singh, 1992).

## Results

### Cross-sectional Effects

**Figure 1:**
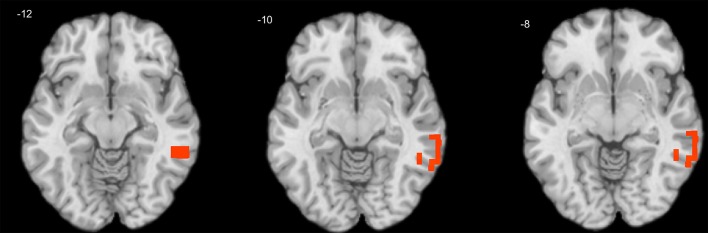
Cross-sectional association of age with theta-beta coupling in children. Warm tints show areas where age correlates positively with the strength of theta-beta coupling.

One-sample *T*-tests showed that only positive correlation coefficients were significant across subjects in both child and adult samples. A series of independent samples *T*-tests revealed no significant gender differences either in personality or EEG variables. In children, the effect of age was significant for theta-beta coupling (max *R* = 0.29, corrected *p* = 0.027), which increased with age in the right middle temporal gyrus (MTG; Figure 1).

#### Adults vs. Children

**Figure 2:**
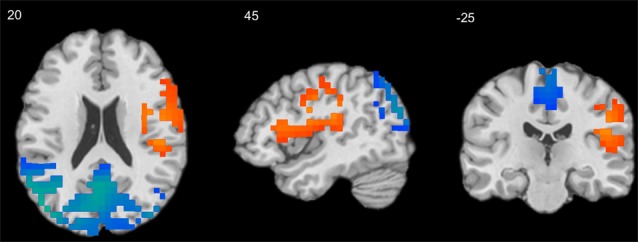
Results of independent-samples *T*-test comparing S-F coupling in children and adults. Warm tints show areas where S-F coupling was stronger in adults, whereas cool tints show areas where it was stronger in children.

A series of independent-samples *T*-tests were performed to compare the strength of delta-alpha, delta-beta, theta-alpha, theta-beta, and mean S-F coupling in children and adults. The critical probability threshold was set at *P* = 0.01, two-tailed, corrected for multiple comparisons. Significant bidirectional (i.e., children > adults and adults > children) effects were obtained in all cases. Because the spatial patterns of these effects were similar for different measures, only the mean S-F coupling results are presented (see Table 2, Figure 2).

**Table 2:** Cross-sectional analysis of mean S-F coupling. Adults < children.

Effect	Area	BA	*x*, *y*, *z*	*t*-value	*p*
positive	Insula	13	45, −25, 20	5.0	<0.00001
	PreCG	6	45, −5, 25	5.0	<0.00001
	PostCG	2	50, −25, 30	5.0	<0.00001
	IFG	44	50, 0, 20	4.6	<0.00001
negative	Precuneus	7	0, −80, 45	−7.1	<0.00001
	SPL	7	−30, −70, 55	−7.0	<0.00001
	IPL	7	−40, −70, 45	−6.7	<0.00001
	AG	39	−50, −70, 35	−6.7	<0.00001
	MTG	39	−50, −75, 25	−6.5	<0.00001

*Note. AG, angular gyrus; IFG, inferior frontal gyrus; IPL, inferior parietal lobule; MTG, middle temporal gyrus; PostCG, postcentral gyrus; PreCG, precentral gyrus; SPL, superior parietal lobule*.

As Figure 2 shows, the strength of mean S-F coupling was higher in children in parietal cortical regions including the precuneus, superior and inferior parietal lobule (IPL), angular gyrus, and posterior part of the MTG, and was higher in adults in the right insula, pre- and post-central gyri, and inferior frontal gyrus (IFG). Additionally, we divided the child sample into three age groups (6–7.5 years, *N* = 57, 7.5–9 years, *N* = 52, and 9–11.5 years, *N* = 61) and compared adults with each one of these groups. Results were very similar for all three comparisons.

#### Effect of Personality in Children

**Figure 3:**
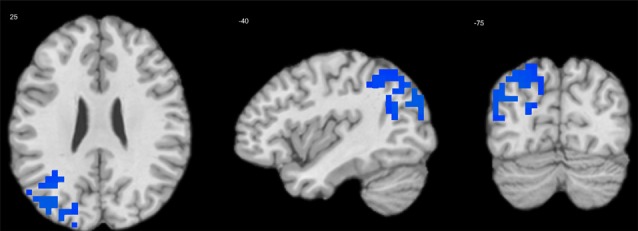
Regression of extraversion scores on delta-alpha coupling in children. Cool tints show areas where extraversion correlates negatively with the strength of delta-alpha coupling.

Extraversion was negatively associated with delta-alpha coupling in the left MTG (BA 39; Table 3, Figure 3). There were no significant effects of other personality variables.

**Table 3:** Effect of extraversion on S-F coupling in children.

Effect	Area	BA	*x*, *y*, *z*	R	*p*
negative	MTG	39	−40, −75, 25	−0.25	0.048

*Note. MTG, middle temporal gyrus*.

### Longitudinal Effects

#### Test-Retest Reliability

An important question is whether S-F coupling measures reflect state-dependent or trait-like properties. To address this issue, the S-F coupling measures were averaged across the 6,430 voxels for each child separately and type C intraclass correlation coefficients (ICCs) using a consistency definition were computed over the four time points. They were 0.76, 0.75, 0.75, 0.78, and 0.79 for delta-alpha, delta-beta, theta-alpha, theta-beta, and mean S-F measures, respectively.

#### Longitudinal Whole-Brain Analysis

**Figure 4:**
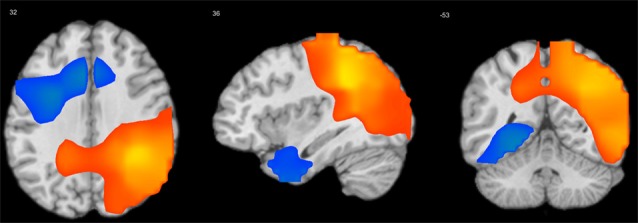
Longitudinal “visit” effect on mean S-F coupling. *p* < 0.05 false discovery rate (FDR-corrected), cluster size >100 voxels. Warm tints show areas where S-F coupling increased with age, cool tints shows areas where it decreased.

Longitudinal effects were analyzed using models consisting of an intercept, the centered mean age per subject $\bar{{\text{Age}}_{i}}-\bar{\text{Age}}$ (referred to as cross-sectional “age” effect), the intra-subject centered age $\bar{\text{Age}}-\bar{{\text{Age}}_{i}}$ (referred to as longitudinal “visit” effect, their interaction (referred to as “acceleration,”) and child’s gender (Guillaume et al., 2014). Only visit effect and acceleration were of interest, all others were used as nuisance covariates. Table 4 presents all effects that were considered significant at *p* < 0.05 (FDR-corrected) and cluster size >100 voxels. Figure 4 shows the longitudinal effect on the mean S-F coupling.

**Table 4:** Whole-brain longitudinal effects.

S-F	Effect	Area	BA	*x*, *y*, *z*	*Z*-value	Q_FDR-corrected_
D-A	Visit+	IPL	40	46, −45, 56	4.96	<0.001
		MTG	21	69, −35, −10	3.95	0.001
D-B	Visit+	IPL	40	38, −50, 47	6.07	<0.001
		IPL	40	−37, −55, 43	3.87	0.001
	Visit−	IFG	45	−60, 30, 0	4.24	0.005
	Accel−	Precuneus	7	10, −80, 45	3.6	0.026
T-A	Visit+	MTG	21	30, −50, 30	6.81	<0.001
		ITG	37	65, −55, −10	6.55	<0.001
	Visit−	ACC	32	−5, 20, 20	7.29	<0.001
		IFG	44	−55, 22, 6	5.99	<0.001
T-B	Visit+	Precuneus	7	25, −45, 45	9.01	<0.001
		IPL	40	30, −40, 40	8.98	<0.001
	Visit−	IFG	45	−50, 30, −20	5.97	<0.001
S-F	Visit+	STG	39	36, −53, 32	6.05	<0.001
	Visit−	IFG	45	−55, 26, −3	5.45	<0.001
		Insula	13	−45, −10, −5	5.08	<0.001
		ACC	32	−5, 19, 30	5.0	<0.001

*Note. ACC, anterior cingulate; IFG, inferior frontal gyrus; IPL, inferior parietal lobule; ITG, inferior temporal gyrus; MTG, middle temporal gyrus; PG, parahippocampal gyrus; STG, superior temporal gyrus; D-A, delta-alpha coupling; D-B, delta-beta coupling; T-A, theta-alpha coupling; T-B, theta-beta coupling; S-F, mean S-F coupling*.

The general pattern of longitudinal changes from age 7–10 years appears to consist of an increase in the strength of coupling in posterior and right-temporal cortical regions and a decrease in left frontal regions. Interestingly, at least for one measure (delta-beta coupling), there was a negative acceleration effect, which implies that the longitudinal increase of coupling in posterior cortical regions was less pronounced in older children. Additionally, we analyzed the interaction of gender with visit effect, but there were no significant interaction effects.

### Individual Growth Curve (IGC) Analysis

For each S-F coupling measure, we extracted values in the voxel that showed maximal positive or, respectively, negative (hereafter positive and negative ROIs) visit-effect in the previously described longitudinal whole-brain analysis (see Table 4), and used them in subsequent linear mixed-effects analyses in SPSS. Individual growth curve (IGC) allows for analyzing irregularly spaced datasets, which is the case here, by using a time-structured predictor (time; Singer and Willett, 2003). For each child, the wave 1 age values were subtracted from his/her age values in all waves. Thus, the wave 1 values of time were effectively set at 0 and the number of months from wave 1 was calculated for each wave of subsequent data collection. Time was added in the model to test the linear effect of time on S-F coupling values. To test a nonlinear developmental trend, higher-order parameter (i.e., Time^2^ and Time^3^) were included. The quadratic time was formed by squaring the linear term and the cubic time was calculated by powering the linear term to three.

An unstructured (UN) residual covariance structure, in which the variance between waves is not constant and the correlations between waves are differed across time, was specified, because it is most commonly found in longitudinal data and requires no assumption in the error structure (Singer, 1998; Shek and Ma, 2011). The linear effect of time was significant for all S-F coupling measures (i.e., it was positive for positive and negative for negative ROIs). Thus, for the mean S-F coupling, significant values in both the intercept and linear slope parameters were obtained for both positive (intercept = 0.25, SE = 0.022, *t* = 10.9, *p* < 0.001; slope = 0.07, SE = 0.01, *t* = 6.6, *p* < 0.001) and negative (intercept = 0.52, SE = 0.027, *t* = 18.8, *p* < 0.001; slope = −0.07, SE = 0.01, *t* = −7.3, *p* < 0.001) ROIs. For the positive ROI, the mean estimated initial value of mean S-F coupling was 0.25 and it increased with time, whereas for the negative ROI, it was 0.52 and it decreased with time.

**Figure 5:**
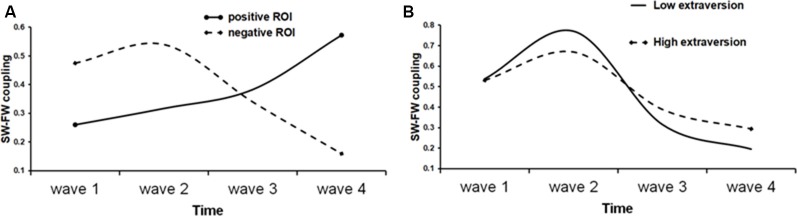
Fitted trajectories of longitudinal change in S-F coupling. **(A)** Solid line shows longitudinal change in “positive” ROI (i.e., ROI that showed general increase of S-F coupling over time), whereas dot line shows longitudinal change in “negative” ROI; **(B)** S-F coupling in negative ROI in low (solid line) and high (dot line) extraversion scorers.

We then proceeded to test the quadratic and the cubic rate of change by adding quadratic or, respectively, cubic parameter in the model. The quadratic trend was significant in all cases and the quadratic model improved model fit over the linear model. Thus, for the mean S-F coupling, the quadratic time trajectories were significant for both positive (estimate = 0.02, SE = 0.007, *t* = 2.52, *p* = 0.013) and negative (estimate = −0.03, SE = 0.008, *t* = −3.6, *p* < 0.001) ROIs. The significant positive quadratic effect for the positive ROI shows that the rate of growth increased over time in this cortical area, whereas the significant negative quadratic effect for the negative ROI is a sign of deceleration. A cubic model was also tested by adding the cubic term to the previous model. The cubic term proved to be significant only for the negative ROIs (estimate = 0.03, SE = 0.009, *t* = 3.3, *p* = 0.001 for the negative mean S-F ROI). The positive effect of cubic growth revealed that the deceleration gradually diminished over time. Figure 5A shows a prototypical plot of longitudinal changes in mean S-F coupling for the positive and negative ROIs.

The fitted trajectories were calculated by using the estimates obtained in the cubic model and substituting the time with average time values. This approach is generally identical to the method of plotting graphs in regression (Aiken et al., 1991) and was used in previous studies (Raudenbush et al., 1995; Cillessen and Borch, 2006).

Finally, we tested whether personality scores may predict the shape of individual growth trajectories. In these analyses, personality scores were treated as a time-invariant continuous covariate to explore any differences in change over time (i.e., interaction with time). Specifically, we examined whether a particular trait was a predictor of the intercept, linear, quadratic, and cubic parameters after controlling the effect of gender and initial age. Parental ratings were available only for the last two waves. They were combined together and averaged if the child had the data for both waves. The resulting sample consisted of 64 children (39 boys). The analyses were performed for all positive and negative ROIs. Significance threshold was set at *p* < 0.01 to correct for multiple tests across the five personality scales. Significant effects emerged only for extraversion and only for negative ROIs. These effects were similar for theta-alpha, theta-beta, and mean S-F coupling, therefore, only the latter will be described. Extraversion was a significant predictor of the linear (*β* = −0.31, SE = 0.11, *t* = −2.8, *p* = 0.006), quadratic (*β* = 0.24, SE = 0.08, *t* = 3.0, *p* = 0.004), and cubic (*β* = −0.04, SE = 0.01, *t* = −2.9, *p* = 0.005) changes in the mean S-F coupling, but was not associated with the initial status (*β* = −0.006, SE = 0.04, *t* = −0.14, *p* = 0.886).

To demonstrate the effect of extraversion on the shape of individual growth trajectories, the sample was divided into high (1) and low (−1) extraversion scorers using the median split and prototypical plots of longitudinal changes in the mean S-F coupling for the negative ROI were constructed for these two groups substituting the respective parameter estimates. Regarding the linear slope, “introverts” showed a faster rate of change as compared with “extraverts” (*β* = −0.31). The shape of the IGC better fitted to quadratic form in “extraverts” than in “introverts” (*β* = 0.24). On the other hand, “introverts” had a faster rate of cubic change than “extraverts” (*β* = −0.04). As Figure 5B shows, generally, the trajectory of longitudinal changes is more stable over time in high than in low extraversion scorers.

## Discussion

In the beginning of the discussion, some general properties of S-F coupling measures should be noted. First, it appears that they are relatively stable over time. Thus, ICC calculated in children over the period of 4 years showed good to excellent consistency of mean S-F coupling measures, implying their trait-like properties. Second, patterns of associations that were revealed for different S-F coupling measures show their general similarity. Differences were observed only in the strength of associations, but not in their direction. In most instances, the summary measure of mean S-F coupling was a good or even the best representative of all coupling measures. This implies that in terms of individual differences and developmental changes, the general strength of S-F coupling is more important than the strength of coupling between specific frequencies. The following discussion will be divided into two sections, i.e., developmental changes and individual differences.

### Developmental Effects

In this study, we have two sources of developmental data, i.e., cross-sectional and longitudinal. Within-group cross-sectional effect of age in children was small and consisted of age-related increase in the strength of coupling between specific frequencies and in specific cortical areas. Cross-sectional between-group effect was strong and included effects in both directions. Cortical areas that showed higher S-F coupling in adults include the primary motor and somatosensory cortex, insula, and the right IFG, which are mostly involved in sensory-motor integration and executive control. Thus, insula is the principal node of the salience network (Dosenbach et al., 2007; Seeley et al., 2007), which implements the stable task control, whereas the right IFG is involved in response inhibition (Menon et al., 2001; Aron et al., 2004; Hampshire et al., 2010), but also in phonological word decisions (Hartwigsen et al., 2010). All these processes strengthen with maturation and are generally more developed in adults than in children (Tamm et al., 2002; Luna and Sweeney, 2004; Rueda et al., 2005).

Cortical areas that showed higher S-F coupling in children (i.e., precuneus, parietal lobule, angular gyrus, and posterior part of the MTG) mostly belong to the posterior hub of the default mode network (DMN), which is active in passive states (Raichle et al., 2001; Raichle and Snyder, 2007). Networks, which increase their activity in attention tasks (e.g., the salience network), are called task-positive networks, whereas DMN is called task-negative network (Fox et al., 2005). Thus, it could be summarized that in adults, S-F coupling is stronger in task-positive brain circuits, which participate in sensory-motor integration, salience detection, response inhibition, and attentional control. Whereas in children, greater S-F coupling is observed in task-negative circuits and, in particular, within the posterior DMN hub, which is involved in self-centered cognition (Vogt et al., 2006).

Our longitudinal data are limited to the period from 7 to 10 years and show that during this time, S-F coupling increases in posterior and right temporal cortical areas, which partly overlap with the areas that were found to have higher S-F coupling values in children than in adults in our cross-sectional analysis. Longitudinal decrease of S-F coupling was found in the left IFG, anterior cingulate, and insula. The posterior part of the left IFG (BA 44 and 45), which is traditionally referred to as Broca’s region, is involved in syntactic computations (Tyler et al., 2011) and is causally involved in reordering during sentence processing (Kuhnke et al., 2017). The anterior cingulate and insula are the principal nodes of the salience network, which has been discussed earlier. Thus, again, these areas are similar to the ones that showed lower S-F coupling values in children than in adults, although in the opposite hemisphere. It appears, therefore, that results of cross-sectional and longitudinal analyses contradict each other if one considers maturation as a linear growth towards the “terminal” adult state.

However, previous developmental studies have shown that growth trajectories are usually nonlinear over time (De Fraine et al., 2005; Greene and Way, 2005). Results of our IGC analysis confirm this notion and show that for both positive and negative ROIs, including quadratic term in the model, improved its fit. Moreover, for negative ROIs, the cubic term also proved to be significant. It is noteworthy that, at least for one S-F coupling measure, a negative effect of the interaction term initial age × visit-effect was revealed, which implies that the longitudinal increase of coupling in posterior cortical regions was less pronounced in older children. This could mean that this increase may take place in a relatively narrow time period coinciding with the beginning of school life and will cease in the subsequent time. Indeed, some evidence shows that the beginning of school life is associated with dramatic changes in cognition (Flavell, 1999), which are accompanied by changes in brain functioning (Knyazev et al., 2017a,b). Overall, it appears that longitudinal S-F coupling changes observed in this study represent just a fragment of a complex developmental trajectory, which could be fully apprehended only on full-scale longitudinal data from early childhood to adulthood.

### Individual Differences

As has been reviewed in the “Introduction” section, different S-F coupling measures, calculated either in between- or within-subject domain, correlate positively with social anxiety and behavioral inhibition or are enhanced both in pathological groups scoring high on social anxiety or showing high salivary cortisol levels, and in experimental situations modeling the state of anxious anticipation. In this study, measures of social anxiety and behavioral inhibition were not available and personality was measured in the framework of the Five Factors Model. Nevertheless, the pattern of associations between S-F coupling and psychometric measures, which have been observed in this study, fits well with the pattern reported in previous studies. Introversion, which is characteristic of individuals with social anxiety disorder (Stein et al., 2001), was associated with enhanced delta-alpha coupling in the left BA 39, also called the temporo-parietal junction, and is involved in processing goals, intentions, and beliefs of others (Saxe, 2006; Van Overwalle and Baetens, 2009). Interestingly, IGC analysis of longitudinal data showed that the amplitude of longitudinal changes in S-F coupling was higher in introverts than in extraverts. Some studies show that S-F coupling increases during stressful social situation in high but not in low socially anxious individuals (Harrewijn et al., 2016). It appears that, overall, S-F coupling is less stable in introverted and socially anxious individuals. In the end, it should be noted that we have not found gender-related differences in S-F coupling measures either in adults or in children and we have not found a mention of such differences in relevant literature. It is possible though that such differences could be revealed in appropriate experimental conditions or in pathological states and this needs further investigation.

### Limitations

One limitation is that we used a limited set of psychometric instruments, which did not include a measure of social anxiety. A limitation of our longitudinal design is that it embraced a limited time span, was unbalanced, unevenly spaced, and lacked longitudinal psychometric data. Besides, four time points are not sufficient for a reliable estimation of higher-order temporal trends. Differences between children and adults should be interpreted with caution, because they may reflect cohort rather than age effect. A limitation of our source localization method is that we used a template head model instead of model based on individual structural MRIs. However, the individual head shape and size were accounted for by measuring the actual position of each electrode.

### Conclusion

This is the first study that explored source-level S-F coupling measures in the developmental perspective using both cross-sectional and longitudinal designs. Our results show that in children, S-F coupling measures demonstrate good consistency over the period of 4 years, implying their trait-like properties. S-F coupling is higher in adults in cortical areas, which participate in sensory-motor integration, salience detection, response inhibition, and attentional control, whereas in children, greater S-F coupling is observed in the posterior DMN hub, which is involved in self-centered cognition. Longitudinal data show that over the period from 7 to 10 years, S-F coupling measures demonstrate nonlinear growth trajectories. It appears that S-F coupling undergoes a complex developmental trajectory with ups and downs associated with different developmental stages. In children, introversion was associated with higher S-F coupling in cortical areas related to emotion, attention, and social cognition. Besides, introverts demonstrate higher amplitude of longitudinal S-F coupling changes. It could be concluded that the association between introversion and S-F coupling appears early in the development and is additionally reflected in specific developmental trends.

## Data Availability

All datasets generated for this study are included in the manuscript.

## Ethics Statement

The study was approved by the Institute of Physiology and Basic Medicine ethics committee. All applicable subject protection guidelines and regulations were followed in the conduct of the research in accordance with the Declaration of Helsinki. The study aim was explained to all adult participants and all parents and they signed the informed consent.

## Author Contributions

GK designed the study, undertook the statistical analysis and wrote the first draft of the manuscript. AS, AB, and ST participated in EEG data collection and analyses. EK, IL, and HS collected and analyzed parental psychometric data and gave feedback to parents. All authors have read and approved the final manuscript.

## Conflict of Interest Statement

The authors declare that the research was conducted in the absence of any commercial or financial relationships that could be construed as a potential conflict of interest.

## Footnotes

^1^http://www.sccn.ucsd.edu/eeglab/
